# Updated EUCAST Clinical Breakpoints against *Aspergillus*, Implications for the Clinical Microbiology Laboratory

**DOI:** 10.3390/jof6040343

**Published:** 2020-12-06

**Authors:** Jesús Guinea

**Affiliations:** 1 Instituto de Investigación Sanitaria Gregorio Marañón, C/ Dr. Esquerdo, 46, 28007 Madrid, Spain; jguineaortega@yahoo.es; Tel.: +34-91-586-7163; 2 CIBER Enfermedades Respiratorias-CIBERES (CB06/06/0058), Madrid, Spain; 3 Servicio de Microbiología Clínica y Enfermedades Infecciosas, Hospital General Universitario Gregorio Marañón, C/ Dr. Esquerdo, 46, 28007 Madrid, Spain

**Keywords:** *Aspergillus*, antifungal resistance, azoles, amphotericin B, EUCAST, clinical breakpoints

## Abstract

Azole resistance poses a problem for the management of patients with invasive aspergillosis. Former species are in fact groups of closely related species (or complexes); cryptic species frequently show high antifungal resistance. The European Committee on Antimicrobial Susceptibility Testing (EUCAST) Definitive Document (E.Def) 9.3.2 includes guidelines for antifungal susceptibility testing on *Aspergillus* spp. and clinical breakpoints for amphotericin B, itraconazole, voriconazole, posaconazole, and isavuconazole against *A. flavus*, *A. fumigatus*, *A. nidulans*, *A. niger*, and *A. terreus*. New clinical breakpoints were released in February 2020 and one of the most relevant modifications was the definition of the new “susceptible, increased exposure” (formerly “intermediate”) category. Another relevant change was the adoption of the concept of area of technical uncertainty (ATU) that refers to problematic areas which involve uncertainty of susceptibility categorisation (e.g., when minimum inhibitory concentrations (MICs) for susceptible and resistant organisms overlap). To accommodate both the new “susceptible, increased exposure” category and the concept of ATU, MICs of azoles and amphotericin B that fall in the former “intermediate” category have been automatically categorized as either R (amphotericin B) or ATU (triazoles). Finally, EUCAST-AFST (Antifungal Susceptibility Testing) decided to adopt new breakpoints for less common species provided that the epidemiological cut-off value (ECOFF) is below or comparable to the breakpoint for the type species (*A. fumigatus*).

## 1. Introduction

*Aspergillus* spp. cause a variety of fungal-related conditions grouped under the term aspergillosis, including allergic manifestations, progressive chronic syndromes such as chronic pulmonary aspergillosis, and the threatening acute invasive infection [1]. *Aspergillus*-related diseases mostly affect different sites of the respiratory tract system, with the bulk of cases involving the lungs. Invasive pulmonary aspergillosis may affect patients with a plethora of underlying health conditions, such as hematologic diseases, chronic lung diseases, patients receiving corticosteroids or monoclonal antibodies, and nowadays patients with post-viral infections such as influenza and COVID-19 [2,3,4,5,6].

Azoles are the drugs of choice for the treatment and prevention of *Aspergillus* infections and are the only available anti-*Aspergillus* oral drugs to date [7,8]. Azoles have different indications for the management of patients with aspergillosis. The European Society of Clinical Microbiology and Infectious Diseases (ESCMID) guidelines recommend itraconazole for patients with allergic or chronic pulmonary aspergillosis. Voriconazole and isavuconazole are indicated as the first-line treatment of pulmonary invasive infections. Posaconazole is recommended for antifungal prophylaxis during prolonged neutropenia or as salvage therapy in patients who are intolerant or do not respond to other drugs. Finally, liposomal amphotericin B is recommended in settings in which azoles are contraindicated because of resistance or intolerance [7].

In a scenario of an increasing number of underlying conditions, the diagnosis and management of aspergillosis has become a challenge. Antifungal susceptibility testing may help guide proper treatment in infected patients. The present manuscript gives an overview of the necessity of performing antifungal susceptibility testing on *Aspergillus* spp. isolates from patients with suspicion of invasive aspergillosis, summarizes available European Committee on Antimicrobial Susceptibility Testing (EUCAST) methodology for that purpose, and includes the latest modifications on azole and amphotericin B EUCAST clinical breakpoints against *Aspergillus* spp. released in February 2020.

## 2. Epidemiology and Patterns of Antifungal Susceptibility of Species Causing Aspergillosis

**Figure 1:**
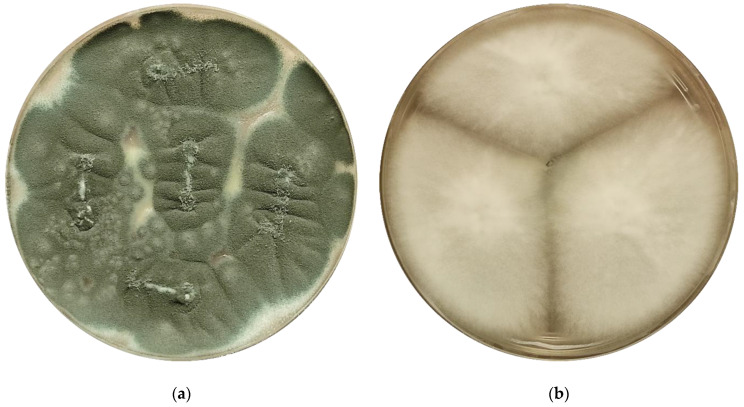
(**a**) *A. fumigatus sensu stricto* and (**b**) *A. lentulus* isolates grown on potato dextrose agar plates.

*A. fumigatus* is the most clinically relevant species causing aspergillosis, followed by *A. flavus*, *A. terreus*, *A. niger*, and other *Aspergillus* spp. [9]. Former *Aspergillus* species are in fact groups of related species that make up 27 complexes or sections [10]. For example, the *A. fumigatus* complex (or *Fumigati* section) includes the dominant *A. fumigatus sensu stricto* species and a number of difficult-to-distinguish species, the so-called cryptic species, that account for 10–15% of isolates within the complex (Figure 1) [11,12].

At the time of writing of this manuscript, the *A. fumigatus* complex included 56 different species [10], *A. fumigatus sensu stricto*, *A. lentulus*, *A. fumigatiaffinis*, and *N. udagawae* being those most commonly found in clinical samples [13]. Identification of isolates of the *A. fumigatus* complex at species level is clinically relevant not only to obtain an insight into the epidemiology, but also because cryptic species commonly show intrinsic resistance to azoles and amphotericin B [14,15]. Years ago, *A. fumigatus sensu stricto* isolates were fully susceptible to azoles, but have been increasingly acquiring azole resistance worldwide after the extensive use of environmental azole fungicides [12,16,17,18].

Other species complexes are intrinsically resistant to polyenes (*A. terreus*, *A. nidulans*, and *A. flavus*) or to azoles (*A. ustus*) [15].

## 3. Clinical Impact of Azole Resistance in the Management of Patients with Invasive Aspergillosis

Primary azole resistance in *A. fumigatus sensu stricto* has commonly been reported in the Netherlands and other Northern Europe countries for the last 15 years. Unfortunately, azole resistance has rippled to many other countries that have ended up being affected to a variable extent [19]. Secondary azole-resistant isolates have been reported in the UK, probably as a consequence of prolonged azole treatment in patients with chronic forms of invasive aspergillosis [20]. A large multicentre study conducted in Spain in 2019 proved that 7.4% of isolates were azole resistant, a higher-than-expected figure in that country [13]. Azole resistance in *A. fumigatus sensu stricto* lies on the presence of point mutations in the *cyp51A* gene; different mutations associate to specific phenotypic resistance to one or more azoles [21,22]. TR_34_-L98H substitutions commonly confer pan-azole resistance; TR_46_-Y121F-T289A usually associate to voriconazole resistance; TR_53_ are rare and result in a phenotype of resistance to itraconazole and voriconazole; finally, a miscellanea of single-point mutations associate to different azole resistance phenotypes [22,23].

Azole resistance in *Aspergillus* spp. isolates remains a clinical problem given the relevant role of triazoles in the management of aspergillosis [7,8,24]. Higher mortality rates have been reported in patients infected by azole-resistant *A. fumigatus sensu stricto* isolates—31% higher day-42 mortality—in comparison to azole-susceptible cases [19,25,26]. Mortality of patients infected with azole resistant isolates is similar to that found decades ago when patients received conventional amphotericin B [18]. In consequence, countries severely affected by the presence of azole-resistant *A. fumigatus sensu stricto* isolates in the environment (>10%) have chosen to prescribe the initially used liposomal amphotericin B or a combination of voriconazole and echinocandins to treat patients, at least until antifungal susceptibility testing results are available [27].

Thus, to improve patient care, tackling azole resistance detection is of paramount importance. The ESCMID 2018 guidelines support identification at a complex level for all clinically significant *Aspergillus* spp. isolates [BIII], and antifungal susceptibility testing of isolates for both clinical management of patients and epidemiological purposes [AII] [7].

## 4. The EUCAST Subcommittee on Antifungal Susceptibility Testing—Methods for the Detection of Azole Resistance in *Aspergillus* spp. Isolates

The Clinical and Laboratory Standards Institute (CLSI) and the EUCAST have proposed conventional methods for the study of azole and amphotericin B susceptibility of *Aspergillus* spp. isolates. The EUCAST Subcommittee on Antifungal Susceptibility Testing (EUCAST-AFST) developed and validated breakpoints and methods for susceptibility testing of yeasts, moulds, and dermatophytes. Among other differences between method in CLSI and EUCAST, the procedures of the latter are freely available online [28]. To date, CLSI does not offer clinical breakpoints against *Aspergillus* spp.

Before conducting antifungal susceptibility testing, isolates must be correctly identified using reliable procedures (MALDI, molecular identification, etc.), at least at complex level. Two methods for testing antifungal susceptibility on *Aspergillus* spp. isolates are available [28]. The EUCAST Definitive Document (E.Def) 10.1 method allows screening for the presence of azole resistance in *A. fumigatus* based on the ability of the isolates to grow on azole-containing agar plates [29]. The procedure was developed for routine use in the clinical mycology laboratory, so that most susceptible isolates are reported as such when no growth is observed on azole-containing agar plates; isolates able to grow on azole-containing plates, and therefore suspicious of being azole resistant, must be confirmed by microdilution methods. The E.Def 9.3.2 method allows the determination of the minimum inhibitory concentration (MIC) of antifungals against *Aspergillus* spp. [30]. Validation of results obtained by either method requires the use of quality control isolates—according to instructions given in the abovementioned documents—whose growth should be inhibited by antifungal MICs within acceptable MICs ranges provided in the v.5 document [31].

As per the E.Def 9.3.2 method, the MIC endpoint for azoles and amphotericin B is the concentration of drug yielding no growth visible to the eye. The procedure recommends ignoring single colonies on the surface of the antifungal-containing wells and “skipped-wells” (growth is seen in alternative wells). However, visual inspection may be challenging and the presence of tiny colonies may raise doubts about MIC interpretation. A recent study compared azole and amphotericin B MICs against *A. fumigatus* complex isolates obtained visually and spectrophotometrically (to overcome subjectivity) [32], showing high agreement between both methods, including for *A. fumigatus sensu stricto* isolates harbouring the dominant *cyp51A* gene mutation. The authors concluded that spectrophotometric MIC reading is a useful alternative to visual inspection and may become an option for determining MIC endpoints of azoles and amphotericin B in the near future.

MICs obtained with the E.Def 9.3.2 procedure should be interpreted using clinical breakpoints/epidemiological cut-off values (ECOFFs), which allows classifying isolates as either susceptible/wild-type or resistant/non-wild-type. Species-specific breakpoints apply to all species within a complex (for example *A. fumigatus sensu stricto* and *A. lentulus*). EUCAST-AFST regularly reviews antifungal clinical breakpoints against *Aspergillus* spp. To date, EUCAST-AFST has developed clinical breakpoints for amphotericin B, itraconazole, voriconazole, posaconazole, and isavuconazole against *A. flavus*, *A. fumigatus*, *A. nidulans*, *A. niger*, and *A. terreus* [33].

## 5. Changes in EUCAST Antifungal Clinical Breakpoints against *Aspergillus* spp. (2020 Update)

Clinical breakpoints were revised and released in February 2020. A recent comprehensive review reports all changes in clinical breakpoints against *Aspergillus* and *Candida* as per the EUCAST methodology [33]. The current manuscript exclusively reviews those for *Aspergillus* spp. The new version format of the clinical breakpoint table in plural was harmonized with the table for antibiotics. One of the most relevant modifications was the definition of the new “I” category, changed from “Intermediate” to “Susceptible, Increased exposure”. The former “Intermediate” category was employed for two reasons: to alert the need of using high antifungal doses to attain enough drug concentrations at the site of infection, and as a buffer zone to prevent small, uncontrolled technical factors that may lead to misclassifications and major discrepancies in interpretation [33]. A test-to-test variation of ± 1 two-fold dilution in the MIC setting is acceptable. To differentiate both scenarios, EUCAST revised the definition of the “Intermediate” category and modified it to “Susceptible, Increased exposure” in cases with high likelihood of clinical success, because exposure to the agent is increased by adjusting the dosing regimen or its concentration at the site of infection [33]. It is noteworthy that the letter “I” was kept, but does not stand for “Intermediate” anymore.

Another relevant modification in the EUCAST breakpoints was the adoption of the concept of area of technical uncertainty (ATU). There are problematic areas that involve uncertainty of susceptibility categorisation that the laboratory should be aware of. EUCAST has identified these and named them ATU (for example when the MICs against susceptible and resistant organisms overlap). The ATU does not refer to unreliable testing procedures and it is assumed that the MIC value obtained is correct. MICs falling in ATU are warnings and should encourage laboratory staff to do “something else” before reporting the MICs as susceptible or resistant, and should only be reported as such to clinicians under special circumstances. ATU was not adopted for the case of amphotericin B. For azoles and *Aspergillus*, MICs falling in ATU should be automatically reported as resistant (itraconazole and voriconazole) or should be deciphered (posaconazole and isavuconazole) based on the susceptibility to other azoles. Instructions to interpret and report MICs in ATU for azoles are given below.

The new EUCAST categories are as follows: S (Susceptible) when there is high likelihood of clinical success using standard doses of the drug; I (Susceptible, Increased exposure) when there is high likelihood of clinical success when exposure to the agent is increased either by adjusting the dosing regimen or by physiological concentration at the site of infection; R (Resistant) when there is high likelihood of clinical failure even when there is increased exposure; and ATU (Area of Technical Uncertainty) to warn laboratory staff of possible difficulties regarding the interpretation of the obtained value. To accommodate the new “I” category and the new concept of ATU, breakpoints for amphotericin B, isavuconazole, voriconazole, and posaconazole against *Aspergillus* spp. have been revisited. For antifungal agents, the new “I” category is only applicable in situations where higher doses of the antifungal drug are needed; however, to date, the EUCAST-AFST has not adopted that category for amphotericin B or triazoles. Consequently, MICs falling in the former “Intermediate” category have been automatically categorized as either R (amphotericin B) or ATU (triazoles).

Finally, EUCAST-AFST decided to adopt new breakpoints for less common species when the ECOFF for the species-drug combination in question is below or equal to the breakpoint for the representative type species (*A. fumigatus* for moulds). Therefore, new breakpoints for isavuconazole, voriconazole, and posaconazole against *A. flavus*, *A. nidulans*, *A. terreus*, respectively, have been set. All the corresponding rational documents have been modified to include the abovementioned breakpoint changes and the new “I” category and ATU definitions.

**Figure 2:**
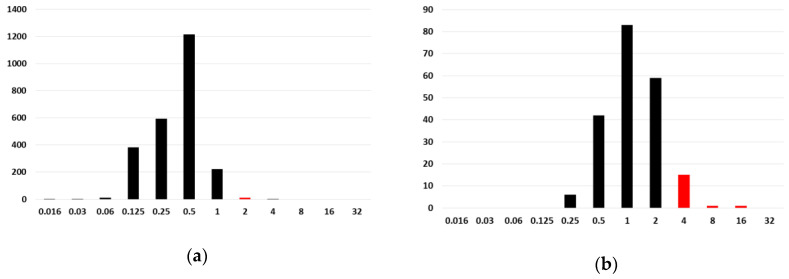

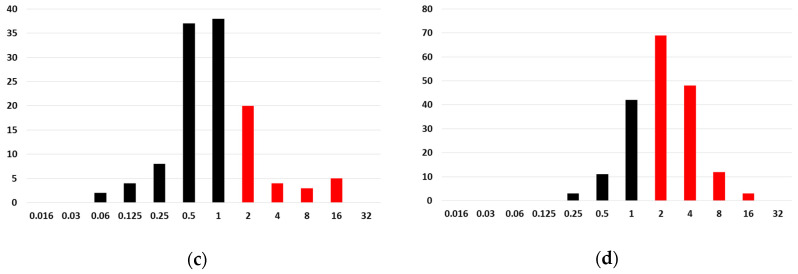
Minimum inhibitory concentration (MIC) distributions of amphotericin B against (**a**) *A. fumigatus*, (**b**) *A. flavus*, (**c**) *A. nidulans*, and (**d**) *A. terreus*. Columns depicted in red and black represent MICs in the area of resistance and susceptibility for *A. fumigatus* isolates, respectively. Data extracted from the amphotericin B rational document v2.0 (https://www.eucast.org/astoffungi/rationale_documents_for_antifungals/, accessed on the 4 December 2020).

The new clinical breakpoints and ECOFFs for amphotericin B against *Aspergillus* spp. are shown in Table 1. Clinical breakpoints have been lowered one-fold dilution against *A. fumigatus* and *A. niger*. Given the high MICs of amphotericin B against *A. flavus*, *A. nidulans*, and *A. terreus*, EUCAST-AFST consider these three species as not good targets for that drug, and no breakpoints are available (Figure 2).

New clinical breakpoints and ECOFFs for itraconazole and posaconazole against *Aspergillus* spp. are shown in Table 2. Isolates with itraconazole MICs falling in ATU (2 mg/L) should be reported as R with the following comment: “In some clinical situations (non-invasive infection forms), itraconazole can be used provided sufficient exposure is ensured”. On the other hand, isolates with posaconazole MICs falling in ATU (0.25 mg/L) should be interpreted either as S, provided that the isolates are also S to itraconazole, and accompanied by the following comment: “Posaconazole MIC is 0.25 mg/L, thus, one dilution above the S breakpoint due to overlapping between wild-type and non-wild-type populations” or as R, provided that the isolates are also R to itraconazole and refer to reference laboratory for *CYP51A* gene sequencing and confirmation of MICs. This was decided because posaconazole resistance in the absence of itraconazole resistance is rare [33].

The new clinical breakpoints and ECOFFs for voriconazole and isavuconazole against *Aspergillus* spp. are shown in Table 3. Isolates with voriconazole MICs falling in ATU (2 mg/L) should be reported as R with the following comment: “In some clinical situations (non-invasive infections forms) voriconazole can be used provided sufficient exposure is ensured”. In contrast, isolates with isavuconazole MICs falling in ATU (2 mg/L) should be interpreted either as S, provided that the isolates are voriconazole wild-type (*A. flavus*: voriconazole MIC ≤ 2 mg/L; *A. fumigatus*: voriconazole MIC ≤1 mg/L), accompanied by the following comment: “The MIC of 2 mg/L is one dilution above the S breakpoint but within the wild-type isavuconazole MIC range due to a stringent susceptibility breakpoint. Please refer to rationale documents for more information”; or as R, provided that the isolates are voriconazole non wild-type and refer to reference laboratory for *CYP51A* gene sequencing and confirmation of MICs. Likewise, isavuconazole resistance in the absence of voriconazole resistance is extremely rare [33].

A recent report of azole resistance in *A. fumigatus* conducted in Spain involved a large number of isolates including some bearing mutations, with most isolates harbouring the TR_34_-L98H substitutions [13]. Resistance rate was calculated using the recently updated clinical breakpoints. The new itraconazole breakpoints clearly separated the mutants from wild-type isolates, as isolates with the TR_34_-L98H substitutions had MICs ≥ 16 mg/L. Likewise, all TR_34_-L98H isolates fell in the category of voriconazole R, although the MIC range was wider for this drug, spanning from 2 mg/L to ≥ 16 mg/L. In contrast, ATU in posaconazole (0.25 mg/L) and isavuconazole (2 mg/L) resulted in the confluence of both TR_34_-L98H isolates (all correctly classified as R) and S isolates [13]. These observations prove the accuracy of the new updated EUCAST breakpoints to separate mutants from wild-type isolates and the ATU as an area of overlap between S and R isolates.

**Figure 3:**
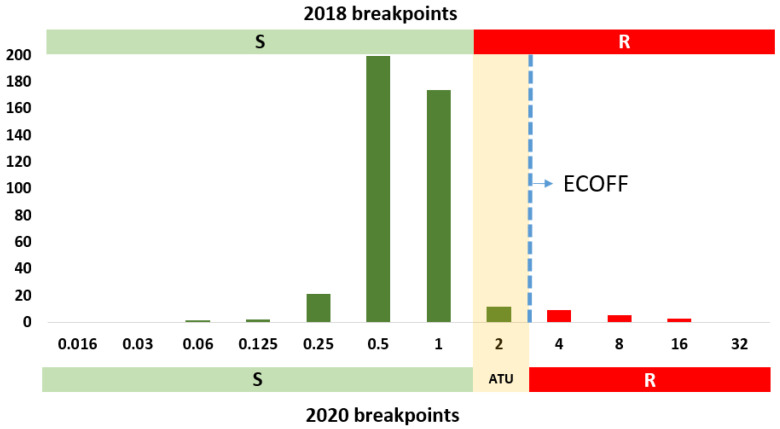
MIC distribution of isavuconazole against *A. fumigatus* complex. Columns depicted in green represent phenotypically isavuconazole wild-type isolates; columns depicted in red represent phenotypically isavuconazole non-wild-type isolates. Horizontal bars indicate clinical breakpoints for the S (green) and R (red) categories; new (2020) and former (2018) breakpoints are shown. The new area of technical uncertainty (ATU) is represented as a light orange shaded column (2 mg/L). Data extracted from the isavuconazole vs. *Aspergillus* rational document v2.0 (https://www.eucast.org/astoffungi/rationale_documents_for_antifungals/ accessed on the 4 December 2020).

**Figure 4:**
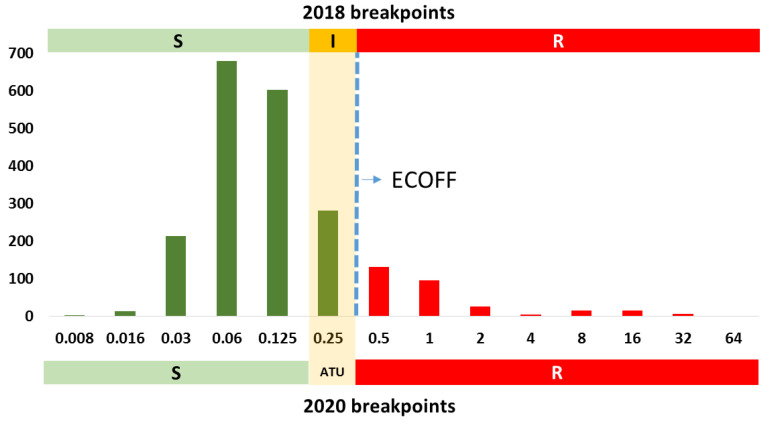
MIC distribution for posaconazole against *A. fumigatus* complex. Columns depicted in green represent phenotypically posaconazole wild-type isolates; columns depicted in red represent phenotypically posaconazole non-wild-type isolates. Horizontal bars indicate clinical breakpoints for the S (green) and R (red) categories, as well as the former “I” (orange) category; new (2020) and former (2018) breakpoints are shown. The new area of technical uncertainty (ATU) is represented by a light orange shaded column (0.25 mg/L). Data extracted from the posaconazole vs. *Aspergillus* rational document v2.0 (https://www.eucast.org/astoffungi/rationale_documents_for_antifungals/ accessed on the 4 December 2020).

Former S breakpoints of isavuconazole (S breakpoint = 1 mg/L and ECOFF = 2 mg/L) and posaconazole (S breakpoint = 0.125 mg/L and ECOFF = 0.25 mg/L) divided the wild-type population because the MIC distribution for wild-type and non-wild-type isolates overlap (Figure 3 and Figure 4).

A stringent breakpoint leads to a number of misclassifications of wild-type isolates as non-susceptible. As an example, a previous study reported 12 isolates with an isavuconazole MIC of 2 mg/L, *cyp51A* gene wild-type sequence, and most of them (9/12 isolates) with a voriconazole MIC ≤ 1 mg/L [34]. However, all isolates had to be classified as resistant as per the former 2018 breakpoints. With the updated 2020 breakpoints, only three out of the 12 isolates are currently voriconazole and isavuconazole resistant, whereas the other nine isolates are fully azole susceptible. Similar observations have been reported elsewhere [35].

## 6. Conclusions

Because of the complexity of patients affected by aspergillosis and the growth in the rate of azole resistance, antifungal susceptibility testing is essential nowadays. EUCAST-AFST have developed procedures for testing antifungal susceptibility in *Aspergillus* spp. and regularly review antifungal clinical breakpoints, the latest update dated February 2020. One of the most relevant modifications was the switch of the former “Intermediate” definition to the new “Susceptible, Increased exposure” category. The letter “I” has been kept in the new category, although it does not stand for “Intermediate” anymore. Another relevant modification is the inclusion of the concept of area of technical uncertainty (ATU). MICs falling in ATU are warnings and should prompt laboratory staff to do “something else” before reporting the MIC as susceptible or resistant. To accommodate the new “I” category and the new concept of ATU, breakpoints for amphotericin B, isavuconazole, voriconazole, and posaconazole against *Aspergillus* spp. have been revisited. To date, EUCAST-AFST has not adopted the new “I” category for amphotericin B or triazoles. Consequently, MICs falling in the former “Intermediate” category have been automatically categorized as either R (amphotericin B) or ATU (triazoles). Finally, EUCAST-AFST decided to adopt new breakpoints for less common species when the ECOFF for the species-drug combination is below or comparable to the breakpoint for the type the representative type species (*A. fumigatus* for moulds). Therefore, new breakpoints for isavuconazole, voriconazole, and posaconazole against *A. flavus*, *A. nidulans*, and *A. terreus*, respectively, have been set.

## Figures and Tables

**Table 1:** ECOFFs and clinical breakpoints for amphotericin B against *Aspergillus* spp. according to the European Committee on Antimicrobial Susceptibility Testing (EUCAST) breakpoint table v 10.0, 2020 [33].

	Amphotericin B (mg/L)		
	WT (ECOFF) ≤	S ≤	R >
*A. flavus*	4	-	-
*A. fumigatus*	1	1	1
*A. nidulans*	4	-	-
*A. niger*	0.5	1	1
*A. terreus*	8	-	-

ECOFF, epidemiological cut-off value; WT, wild-type; S, susceptible; R, resistant; ATU, area of technical uncertainty; -, not good targets for amphotericin B.

**Table 2:** ECOFFs and clinical breakpoints for itraconazole and posaconazole against *Aspergillus* spp. according to the EUCAST breakpoint table v 10.0, 2020 [33].

	Itraconazole (mg/L)				Posaconazole (mg/L)			
	WT (ECOFF) ≤	S ≤	R >	ATU	WT (ECOFF) ≤	S ≤	R >	ATU
*A. flavus*	1	1	1	2	0.5	ND	ND	ND
*A. fumigatus*	1	1	1	2	0.25	0.125	0.25	0.25
*A. nidulans*	1	1	1	2	0.5	ND	ND	ND
*A. niger*	4	ND	ND	ND	0.5	ND	ND	ND
*A. terreus*	0.5	1	1	2	0.25	0.125	0.25	0.25

ECOFF, epidemiological cut-off value; WT, wild-type; S, susceptible; R, resistant; ATU, area of technical uncertainty; ND, not defined.

**Table 3:** ECOFFs and clinical breakpoints for voriconazole and isavuconazole against *Aspergillus* spp. according to the EUCAST breakpoint table v 10.0, 2020 [33].

	Voriconazole (mg/L)				Isavuconazole (mg/L)			
	WT (ECOFF) ≤	S ≤	R >	ATU	WT (ECOFF) ≤	S ≤	R >	ATU
*A. flavus*	2	ND	ND	ND	2	1	2	2
*A. fumigatus*	1	1	1	2	2	1	2	2
*A. nidulans*	1	1	1	2	0.25	0.25	0.25	ND
*A. niger*	2	ND	ND	ND	4	ND	ND	ND
*A. terreus*	2	ND	ND	ND	1	1	2	ND

ECOFF, epidemiological cut-off value; WT, wild-type; S, susceptible; R, resistant; ATU, area of technical uncertainty; ND, not defined.
